# Exploiting rotational asymmetry for sub-50 nm mechanical nanocalligraphy

**DOI:** 10.1038/s41378-021-00300-y

**Published:** 2021-10-20

**Authors:** Nikolaos Farmakidis, Jacob L. Swett, Nathan Youngblood, Xuan Li, Charalambos Evangeli, Samarth Aggarwal, Jan A. Mol, Harish Bhaskaran

**Affiliations:** 1 Department of Materials, University of Oxford, Parks Road, Oxford, OX1 3PH UK; 2 Department of Physics, Queen Mary University of London, London, E1 4NS UK

**Keywords:** Electrical and electronic engineering, Electronic properties and materials, Electronic devices

## Abstract

Nanofabrication has experienced extraordinary progress in the area of lithography-led processes over the last decades, although versatile and adaptable techniques addressing a wide spectrum of materials are still nascent. Scanning probe lithography (SPL) offers the capability to readily pattern sub-100 nm structures on many surfaces; however, the technique does not scale to dense and multi-lengthscale structures. Here, we demonstrate a technique, which we term nanocalligraphy scanning probe lithography (nc-SPL), that overcomes these limitations. Nc-SPL employs an asymmetric tip and exploits its rotational asymmetry to generate structures spanning the micron to nanometer lengthscales through real-time linewidth tuning. Using specialized tip geometries and by precisely controlling the patterning direction, we demonstrate sub-50 nm patterns while simultaneously improving on throughput, tip longevity, and reliability compared to conventional SPL. We further show that nc-SPL can be employed in both positive and negative tone patterning modes, in contrast to conventional SPL. This underlines the potential of this technique for processing sensitive surfaces such as 2D materials, which are prone to tip-induced shear or beam-induced damage.

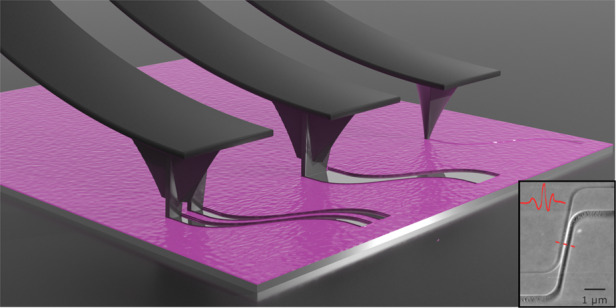

## Introduction

The remarkable properties of nanostructured particles and surfaces such as quantum dots^1^, nanotubes^2^, and metasurfaces^3^, have enabled the development of devices with unprecedented performance and functionality. Continued progress in nanotechnology however, relies on our ability to effectively define structures with 1–100 nm precision on a variety of materials^4–6^; a task which becomes increasingly challenging as device dimensions shrink and the range of materials expands. To achieve this increasingly onerous task, a wide spectrum of top-down and bottom-up approaches have been deployed with emphasis on resolution, cost, and throughput—depending on the application—and ranging from complementary metal-oxide-semiconductor (CMOS) processes for nanoelectronics^7,8^ to the additive patterning of biological^9,10^ and 2D materials^11–14^.

Among these methods, the family of scanning probe lithographies (SPLs) have attracted significant attention due to their unmatched flexibility in materials processing, maskless operation, and the ultra high resolution they afford^5^. In particular, the atomic force microscope (AFM) has accelerated the development of a multitude of diverse imaging and patterning modes driven by mechanical^15,16^, thermal^17,18^, or electrochemical^19–22^ forces and includes both conventional top-down and exotic bottom-up approaches^8,11,23–26^. The collocation and seamless interchangeability between the imaging and patterning modes in an AFM, in conjunction with the ability to operate under ambient or near-ambient conditions, has significantly expanded the choices of materials to include hydrogels, self-assembled monolayers, and liquids, leading to the emergence of a wide spectrum of novel devices ranging from organic electronics to sensors^27–29^.

These attributes, in principle, render AFM-based patterning an ideal process for prototyping nanoscale devices with high resolution and at low cost. However, while AFM-based patterning shows promising results for 0-D (dots) and 1-D (lines) patterning, tip-induced artifacts tend to arise frequently when defining 2-D geometries^30^. Furthermore, the patterning speed associated with SPL becomes a significant obstacle when transitioning to 2-D patterning, where the lithography time scales approximately inversely with the square of the patterning resolution^31–33^. While the patterning speed has been addressed mostly through parallelization^34,35^, the inability to control the resolution in real time, which is invariantly determined by the geometry of the tip’s apex, leads to a rigid constraint of throughput to the resolution required by several critical dimensions in the design regardless of their abundance. This drawback is particularly limiting in SPL, as it has long been addressed in charged beam nanofabrication methods, where control over the aperture size and the particle flux, as well as beam shaping, allows for a resolution that is tailored to specific parameters of the design^36,37^.

The aforementioned limitations associated with throughput and reliability in 2-D patterning have significantly hindered the wider adoption of SPL, making electron beam lithography (EBL) and photolithography (PL) the go-to processes for most research and commercial applications. In this work, we address the disproportional time penalty of an incremental increase in resolution by developing processes that resolve the trade-off between the resolution and reliable large-scale patterning. In particular, we find that these issues can be addressed by breaking the rotational symmetry of the tip, which results in a directional dependence on patterning and imaging. Herein, we demonstrate that this effect can be leveraged to control the resolution in situ and in real time with high fidelity. Using this technique, which we term nanocalligraphy scanning probe lithography (nc-SPL) due to its similarities with conventional calligraphic writing, we unlock an additional degree of freedom found in the patterning direction, giving us the ability to define both micron and nanometer-scale features on a single scan path. By fabricating custom asymmetric AFM tips for mechanical nanolithography and simulating their angular dependence in patterning, we determine the precise directional path required to achieve a desired set of patterns with a given tip. In this way, we achieve an increase in throughput of more than four orders of magnitude in certain cases, which results from a) 100–1000 fewer passes for the definition of large features and b) 10–100 times faster writing speeds afforded by the rigidity of nonapex-like tips. Importantly, we demonstrate minimal tip wear, as evaluated by scanning electron micrographs (SEM) collected before and after patterning of more than 10^4^ μm^2^ through 250 nm of resist using the same tip (Fig S2).

## Probe fabrication

**Fig. 1:**
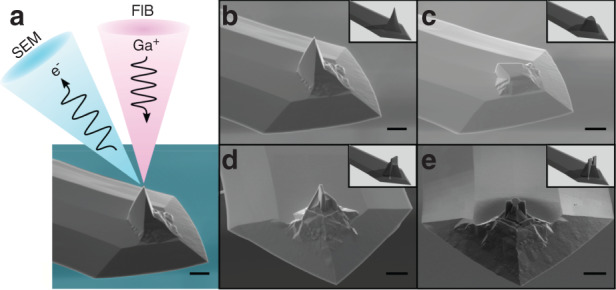
Introduction of rotational asymmetry in custom AFM probes. **a** Graphical illustration of simultaneous milling via FIB and imaging using SEM. **b** Tapping-mode AFM tip prior to milling. **c–e** Plateau, chisel, and double-tip. The asymmetry of these designs can be leveraged for the fabrication of highly complex patterns via mechanical nanolithography. Insets of Figures **b–e** correspond to graphical renderings of the probe geometry. Scale bars are 5 μm

Conventional fabrication of AFM probes at scale typically affords little room for customization since the shape of the tip is primarily determined by the material properties and crystal orientation of the substrate and secondarily on the lithographic operations performed on the substrate^38^. While limited work on novel geometries has been explored and some scalable techniques have been developed^39–41^, we find that more exotic tip geometries and the capabilities that they may enable have yet to be extensively explored and harnessed. Here, we opt to directly modify conventional AFM probes using a Ga^+^ focused ion beam (FIB); this enables us to readily create a variety of novel structures at the tip, albeit with some limitations in throughput and resolution which are offset by the prototyping flexibility that direct milling affords. Through FIB milling with in situ SEM feedback, we create asymmetric and multiple-tip geometries that are then utilized for direction-dependent patterning (Fig. 1a).

In particular, we have focused on two novel types of tips, which we term “chisel-tip” and “double-tip” configurations, which show particular promise for nc-SPL. These types of tips are produced by sequentially milling a conventional AFM tip (Fig. 1b) into plateau tips (Fig.1c). Plateau tips are subsequently further milled into chisel-tip or double-tip configurations depending on the application; two examples of these types of tips are shown in the SEM micrographs of Fig. 1d and e, respectively. The profile of the chisel-tip and double-tip configurations can be seen in the insets of Fig. 1d and e, respectively, while a detailed procedure for the production of these probes can be found in supplementary section S1.

### Chisel-tip lithography

Conventional AFM probes, which commonly serve the dual purpose of both the lithography and imaging tools, consist of a cantilever beam that dictates the mechanical response of the probe and a pyramid-like tip with a sharp apex (Fig. 1b). The sharpness of the tip, commonly defined by a corner radius at the apex, thus determines an upper bound for the resolution achievable. In contrast to conventional AFM probes, the chisel tip is designed to have a straight, rectangular profile with asymmetric length (*l*) and thickness (*t*). While the apex of a conventional AFM probe is rotationally symmetric and an identical linewidth is produced when the probe is scanned in any arbitrary direction, a chisel-tip with a rectangular cross-section will produce lines of different widths (*w)* depending on the direction it is scanned.

**Fig. 2:**
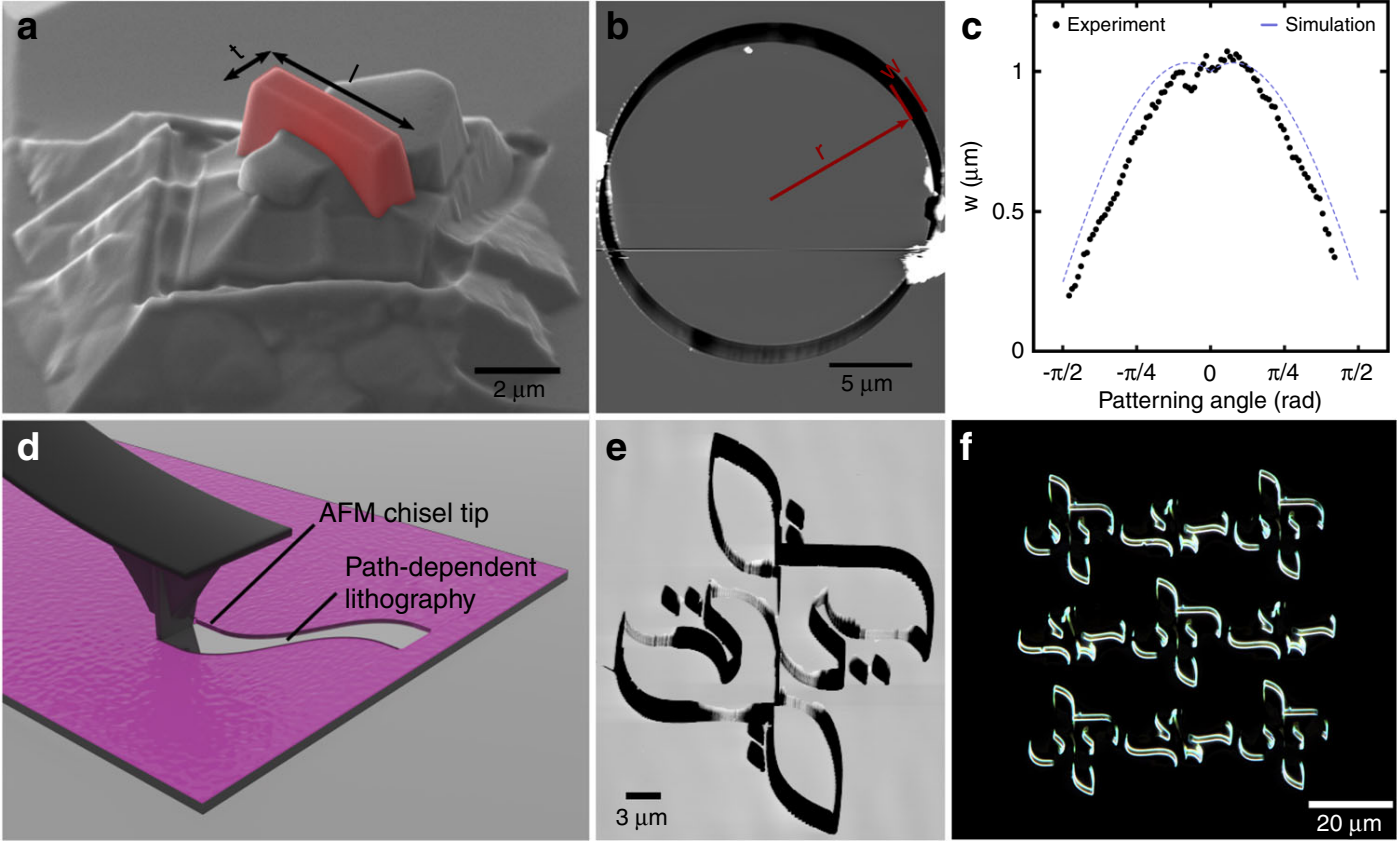
Exploiting rotational asymmetry of a chisel-tip. **a** False-color SEM micrograph of a typical chisel tip. **b** AFM micrograph of circle produced on PMMA. **c** Simulation results indicating the patterned linewidth as a function of the patterning direction shown in blue. The experimentally determined linewidth is plotted alongside in black. **d** Graphical illustration of the directional dependence of the produced lithography. **e-f** AFM and dark-field optical micrographs of complex patterns produced using a single, continuous probe trajectory. Artwork is by calligraphy artist Majid Alyousef

This effect can be accurately simulated for different dimensions *w* and *t* by parametrizing the profile of the tip and determining the effective cross section (ECS) when scanned at different angles relative to the longitudinal axis of the probe. By superimposing the tip’s cross-section on the trajectory followed by the probe, the precise output can be determined. These simulations allow us to predict the lithography result and optimize the tip scan path until a close match is found between the desired features to be produced and the patterned features. Importantly, the linewidth produced scales with both the trajectory followed by the probe and the dimensions (*l)* and (*t)* of the tip. The dependence of the simulated linewidth (*w)* on the patterning angle for increasing probe length *l* can be seen in Fig. S5. Here, each of the solid lines corresponds to a different chisel length between 0.5 μm and 5 μm simulated between -π/2 and π/2 radians. The thickness of the tip was fixed to 0.25 μm, as the aspect ratio of the width to the length of the tip provided sufficient angular dependence for patterning and simultaneously provided enough support to the probe for high durability. We evaluate the accuracy of the simulated results by employing a chisel-tip with *l* = 1 μm and *t* = 250 nm (Fig. 2a) to produce a circle with a radius of *r* = 10 μm on 495k poly(methyl methacrylate) (PMMA) EBL resist, as shown in Fig. 2b. The linewidth of the circle produced is then imaged using conventional AFM and is measured perpendicular to the lithography path. The linewidth is then compared to the simulation results for the specific probe dimensions. The measured linewidth shown in Fig. 2c shows excellent agreement with simulations both qualitatively and quantitatively, with a minimum width of 200 nm and maximum width of 1.1 μm.

**Fig. 3:**
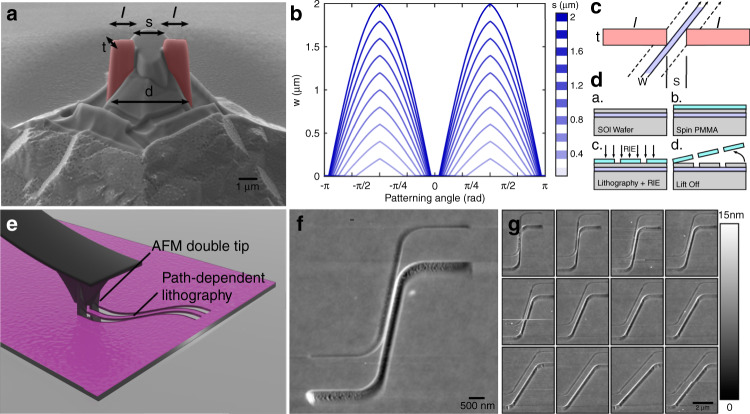
Exploiting rotational asymmetry of a double-tip probe. **a** False-color SEM micrograph of a typical double-tip. **b** Simulation results indicating the patterned linewidth as a function of the probe geometry and patterning direction. **c** Plan view schematic of the dependence of the two patterned paths from each of the two tips. The distance between the two paths is directly correlated to the patterning direction and the geometry of the double-tip. **d** Schematic of sequential schematic of wafer stack and pattern transfer to the substrate. (a) Initial stack, (b) spin coating of PMMA thin film, (c) pattern transfer by RIE, (d) lift-off, and **e** graphical illustration of the directional dependence of the produced lithography. **f**, **g** AFM micrographs of patterns transferred onto an SOI wafer. The patterning angle is varied between 2.5 and 30° with an increment of 2.5°. The width of the resulting nanowires was varied between 1 μm and sub-50 nm as a result of the changes in the patterning angle. All patterns were written with the same tip, and only the patterning angle was varied, with all other parameters remaining unchanged

Owing to the asymmetry of the probe, complex lithographic patterns of different dimensions can be produced, allowing for intricate patterns without scanning artifacts or resist buildup at the borders of the lithography path. This effect is illustrated in Fig. 2e and e, where arbitrary calligraphy patterns are made using this technique (reproduced with permission from calligraphy artist Majid Alyousef). A cross section of the pattern can be seen in Fig. S6. Using a simulation algorithm wherein the scanning direction of the probe is calculated at each point of the design and subsequently substituted by the linewidth calculated in each direction, we are able to predict the expected lithography pattern with high precision and match the desired design. Thus, we are able to reproduce complex features with a variety of probe shapes and a high degree of continuity. Minimizing the error between the desired shapes and the simulated output allows us to recreate patterns with high fidelity, as seen in the AFM micrograph and dark-field optical microscope image in Fig. 2d and e, respectively. The patterns shown in micrographs 2D and 2E were transferred to the oxide layer of the substrate via CHF_3_ reactive ion etching, further demonstrating the utility of this technique in pattern transfer (Fig. 3d). Two important advantages of this approach are a) the patterning speed and b) the longevity associated with nonapex-like tips.

One limitation of SPL is its inherently low throughput and the high wearing rate of sharp tips^42^. We address these limitations using asymmetric probes, where precise control over the scanning angle can result in large and fine structures in a single patterning step; this reduces the total writing time by three orders of magnitude in some cases, depending on the pattern density and configuration. Here, the write-time improvement is calculated by finding the path length difference required to fill a pattern using the chisel tip that we employ and a conventional AFM tip with a tip diameter equal to the minimum feature size of the lithography. Further, our chisel-tips are found to retain their integrity, as virtually no damage is observed on the probe before and after patterning several thousand μm^2^ of patterns through more than 250 nm of resist (see section S2 for pre- and postpatterning SEM micrographs). We attribute this considerable increase in tip longevity to the larger cross section of the probe, which has been shown to help reduce tip wear^43^. This increase in structural integrity not only opens up opportunities for high-speed AFM patterning but also enables a wider variety of resists to be used, enabling more flexibility in SPL patterning.

### Double-tip lithography

Our patterning results obtained using a chisel-tip demonstrate the ability to both precisely tailor the linewidth based on the patterning angle and to predict and match complex patterns. This technique also results in vertical sidewalls while having low tip wear. However, a frequent requirement in nanopatterning is for the critical dimensions for devices to be below a few nanometers. Frequently, a nanoscale gap or pattern is required, such as two conducting lines close to each other without overlap or a nanogap without a short^44^. To achieve this, we explored another asymmetry of the tip using a double-tip configuration. In this configuration, two identical tips are produced with a separation (*s)* between them. A false-color SEM micrograph can be seen in Fig. 3a, which represents a double-tip configuration with probe width (*t)*, separation (*s)*, and total length (*l)*. As in the chisel-tip configuration, individual paths can be simulated, and the distance between them as a function of the patterning angle can be extracted. Simulated results for double-tip configurations for different separations (*s)* and a fixed thickness of 250 nm can be found in Fig. 3b. Furthermore, a schematic in Fig. 3b depicts the dependence of the space *w* between the two paths on the patterning angle. The distance between the paths scales similarly to a chisel probe owing to its asymmetric nature but because of the presence of two separate asperities, there is a gap between the center of the pattern. This gap depends on the direction of the scanning of the probe, as illustrated in Fig. 3e.

It is important to note that the individual paths of the two tips moving simultaneously overlap at a critical angle (*α*), where instead of two separate paths, the two converge into a single, wider path. It is thus evident that the double-tip configuration offers the theoretical possibility for infinitesimal resolution in patterning. In practice, however, factors, such as imperfections of the fabricated tips, resist limitations, and imperfect control in the motion of the probe limit the ultimate resolution of this method.

We then use such a double-tip to pattern constrictions of varying dimensions while retaining micron-sized pads, which can be used for electrical connections. As seen in the AFM micrographs of Fig. 3g and f, constrictions ranging between 50 nm and 1 μm can be produced by a single path using a double tip. These patterns were produced at a speed of 20 μm/s and with the same tip, which showed no apparent wear after patterning, as shown in supplementary S2. Thus, we demonstrate that precise simulation and patterning at high resolution is afforded via the double-tip configuration, enabling continuously varying features down to the nanoscale. This is unprecedented resolution between the lines in SPL, using tips that do not necessarily have nanoscale dimensions themselves, thus allowing us to pattern gaps that are effectively much smaller than the minimum probe dimensions. For example, in Fig. 3g, the minimum feature size achieved is 50 nm using a double-tip, where each tip has a length of ~1 μm and a separation of ~2 μm. This is a 50-fold improvement in resolution over the tip dimension, allowing for such patterns to be created.

### Nanolithography corrections

One important attribute of SPL is the colocation of patterning and imaging modes, which permit imaging the substrate without modifying the resist layer and to subsequently perform the required lithography. This capability enables the precise alignment and overlay of the desired features to pre-existing structures on the substrate without modifying the resist, as would be the case if imaging was performed using a charged beam, which would locally expose the resist. Additionally, SPL permits alignment to underlying features that are sensitive to charged beams.

**Fig. 4:**
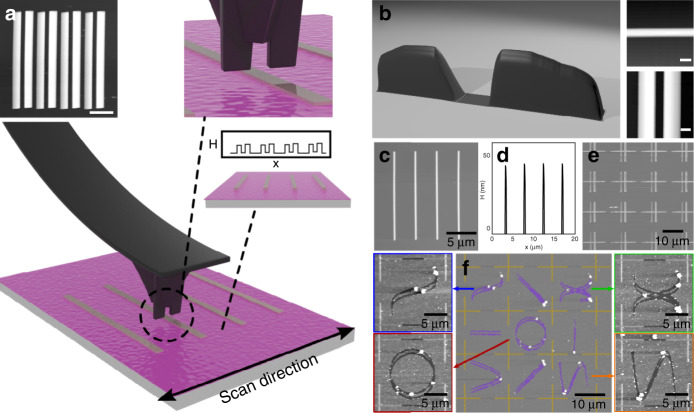
Probe reconstruction, axial rotation artifacts, and pattern overlay. **a** AFM micrograph produced by imaging rectangular nanopillars scanned in the direction perpendicular to their length. **b** Orthographic-view reconstruction of the double tip by sequential AFM imaging nanopillars at 0° (inset bottom) and 90° (inset top), scale bar is 1 μm **c** AFM micrograph of 250 nm wide ridges patterned by EBL and imaged using a conventional tip. **d** AFM section plot of the ridges in (c). **e** AFM micrograph of alignment markers collected using a double tip before patterning. **f** AFM micrographs collected using a conventional tip after patterning thereby demonstrating the ability to overlay patterns in nc-SPL

To maintain this feature of SPL when using our chisel- and double-tips, we devise a method for in situ imaging of the precise structure of the probe. This is achieved by using a calibration sample consisting of ribs and pillars 250 nm in width, as seen in the micrograph of Fig. 4c, as well as the AFM section plot in Fig. 4d. Since the topography of these structures is known, we can use this information to scan the tip over the calibration sample in two orthogonal dimensions and use the deflection information from the AFM to reconstruct the tip geometry. An illustration of this procedure can be seen in Fig. 4a. Furthermore, Fig. 4b shows two AFM micrographs of a calibration sample consisting of a patterned rib when imaged using a double tip at 0 and 90 degrees. Postprocessing these micrographs results in the reconstruction of the probe, as shown in the 3D double-tip rendering of Fig. 4b. This method, which is generalizable beyond the double-tip geometry, permits the characterization of a wide variety of tips and can be used to correct for any tip imperfections. Information obtained by employing this process, coupled with probe profile parameterization, allows for the patterning of complex geometries with intricate tips and could even be used to compensate for any tip variability over time.

We further demonstrate that we can use the double-tip to image pre-existing cross-shaped registration markers on the substrate in contact mode, as shown in the micrograph of Fig. 4e. Using the information on the probe geometry and the scan of the markers, we demonstrate that precise alignment and pattern overlay are achieved. Fig. 4f shows the precise positioning of arbitrary patterns to the cross-shaped registration markers.

**Fig. 5:**
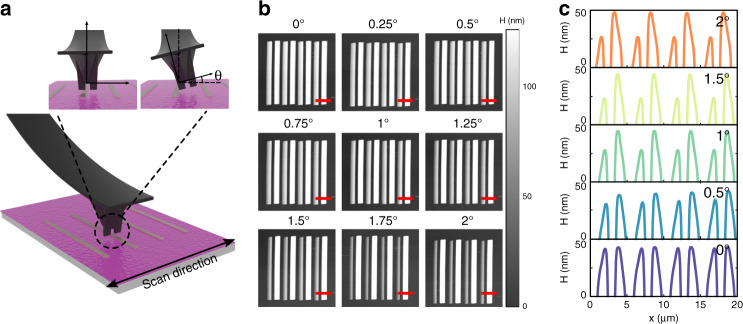
Correction of the probe rotational misalignment resulting from tilting the probe along the axis of the cantilever. **a** Schematic illustrating a perfectly aligned probe and a rotationally misaligned probe by an angle θ. **b** AFM micrographs of 250 nm ridges imaged in contact mode using a double-tip for increasing tilt angles. It is apparent that as the probe becomes tilt, one of the two probes increasingly loses contact with the substrate, and the scale bars are 5 μm. **c** Sections of the micrographs in **b** showing the change in the height measured by each of the two tips

The larger dimensions of nc-SPL probes compared to those of conventional AFM probes, with typical radii on the order of 10 nm, make them more sensitive to rotational misalignment (θ). It is therefore important that an additional calibration step be performed prior to patterning, with the aim of correcting for the tilt of the probe, as illustrated in Fig. 5a. In particular, as seen in the micrographs of Fig. 5b, a tip that is misaligned by 1–2° will result in one of the two tips being 25 nm higher than the other. While we find that improper rotation of the probe can result in patterning artifacts, proper alignment can account for errors in the fabrication of double-tip AFM probes.

## Conclusion

We have developed and demonstrated nanocalligraphy scanning probe lithography using probes with asymmetric tip geometry and by exploiting their rotational asymmetry. We demonstrate nc-SPL by showing its application to mechanical scanning probe lithography by patterning complex lithographic designs. We then demonstrate that resolution is no longer restricted by the size of the tip. We are able to pattern sub-50 nm constrictions with excellent control over their dimensions. nc-SPL can pattern both large and small features using a single lithography step, with insignificant tip wear and vertical sidewalls and up to 10,000 times higher throughput. We have also shown that we can utilize dynamic geometry reconstruction and use this to generate complex patterns. Our technique is readily extended to electrical, thermal, or additive manufacturing lithography methods.

## Materials and methods

### Probe Fabrication

Conventional tapping-mode AFM probes (Tap300-G) with a resonance frequency of 300 kHz were purchased from Windsor Scientific Ltd. The high spring constant (≈40 N/m) of these probes produces small vibrational deflections when milling them and is therefore preferred to soft-tapping or contact mode probes. The probes were subsequently loaded to Zeiss Auriga Ga^+^ FIB-SEM with ATLAS nanopatterning software. A bulk milling step was first conducted parallel to the plane of the cantilever to produce a plateau tip. Subsequently, the tips were rotated 90°, and the desired geometry was milled onto the plateau tip, as illustrated in Fig. S1. Typical milling parameters for the production of the plateau tips include an initial probe current of 500 pA-1 nA for the bulk mill to produce the plateau, and subsequently, the more delicate production of the double-tip and chisel-tip geometries was performed with sub-100pA probe currents. All milling was done with an accelerating voltage of 30 kV.

### Substrate preparation

P-doped <100> silicon wafers with 3.3 μm of wet thermal oxide were purchased from Inseto Ltd. Following conventional processing in acetone and rinsing in isopropyl alcohol, the wafers underwent a cleaning step using a heated piranha solution of 5:1 H_2_SO_4_:H_2_O_2_ to 60 °C for 10 min, as well as an O_2_ plasma clean for 3 min at 200 W. The wafers were then spin-coated with a solution of poly(methyl methacrylate) PMMA 495k with 4% solids in anisole at 6000 rpm and baked for 10 min at 180 °C. Identical processing steps were followed for the patterning of silicon on insulator substrates (SOI) for the production of nanowires with a 1% solution. The wafers in this case consisted of a p-doped carrier layer, 300 nm of wet thermal oxide, and 75 nm of undoped silicon.

### Lithography and imaging

Custom chisel-tip and double-tip probes were loaded onto an Asylum MFP3D AFM in contact mode. A calibration sample including 250 nm wide ridges was subsequently loaded to assist with the rotational alignment of the tip. The sample was then iteratively scanned, and the rotation of the head was adjusted to achieve optimal contact of the entire surface of the tip with the substrate. As a final alignment step, the calibration sample was scanned at 0° and 90°, and the images were postprocessed to determine the precise 3D geometry of the tip. The acquired tip information was fed into a custom code to determine the required offset for alignment to pre-existing features on the substrate to be patterned. The calibration sample was next exchanged with the resist-coated sample to be patterned. The sample was imaged using double-tip or chisel-tip probes depending on the application, and the offset was applied to the pattern designer. Finally, the desired lithography output was processed using the probe information using the algorithm described in supplementary S2 and patterning followed by a force of 1 μΝ at a patterning speed of 10 μm/s.

### Pattern transfer

For the pattern transfer of the lithography to the substrate, we demonstrated both metal deposition via thermal evaporation and reactive ion etching. Prior to deposition, a brief 30-second UV-ozone plasma clean was performed to ensure adhesion to the substrate. This step is used to remove residual traces of photoresist on the surface prior to pattern transfer. Subsequently, the substrates were loaded in an evaporation chamber where an adhesion layer of 5 nm chromium and 30 nm gold were deposited. For the patterns transferred via reactive ion etching, the patterned substrates were directly loaded into the chamber and were etched for 30 s using 100 ccpm CHF_3_ at 200 W.

## Supplementary information

Supporting Information
