# Supplementary material for: Exploiting rotational asymmetry for sub-50 nm mechanical nanocalligraphy
Source: Microsyst Nanoeng. 2021 Oct 20;7:84. doi: 10.1038/s41378-021-00300-y (PMC8528849; doi:10.1038/s41378-021-00300-y)
Supplement: Supplementary file 1 — Supporting Information [file 41378_2021_300_MOESM1_ESM.docx]

**Exploiting rotational asymmetry for sub-50nm mechanical nanocalligraphy**

*Nikolaos Farmakidis^1^*^†^*, Jacob L. Swett^1^*^†^*, Nathan Youngblood^1^, Xuan Li^1^, Charalambos Evangeli^1^, Samarth Aggarwal^1^, Jan A. Mol^1,2^ and Harish Bhaskaran*^1^*

^1^Department of Materials, University of Oxford, Parks Road, Oxford OX1 3PH, UK.

^2^Department of Physics, Queen Mary University of London, London E1 4NS, UK.

^†^These authors contributed equally to this work.

*Corresponding authors: E-mail: *harish.bhaskaran@materials.ox.ac.uk*

Keywords: Scanning-Probe Lithography, Nanomanufacturing, Atomic Force Microscopy, High Resolution

**S1. Tip Fabrication**

**S2. Damage Evaluation**

**S3. Pattern Designer**

**S4. Pattern Transfer**

**S5. Effect of Chisel-Tip Geometry to Linewidth**

**S6. Section Height and Pattern Roughness**

**S1. Tip Fabrication**


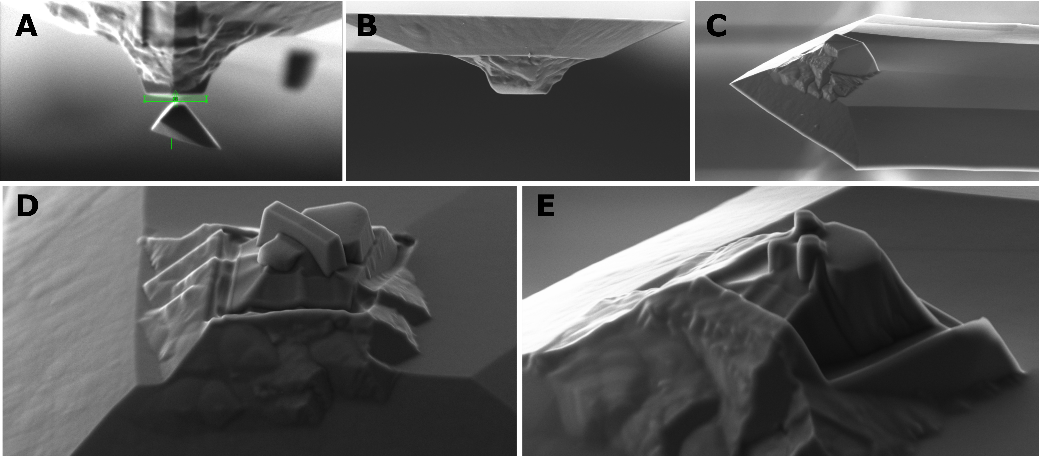
The fabrication process of the custom chisel and double-tips begins by performing a section along the plane of the cantilever. Figure S1A demonstrates a tip which is undergoing FIB milling and the tip is is beginning to detach from the probe. After having completed the section, what remains is referred to as a plateau tip which can be seen in figures S1B and S1C. The plateau tip, which serves as the starting point for all tips we fabricated is subsequently further milled into a chisel-tip or a doubletip depending on the application. In both cases, the tip is produced by a subtractive process which digitally masks an area, and then FIB milling the material around the masked area. Chisel and double tips fabricated using this procedure can be seen in figure S1D and S1E respectively. In spite of broad beam width and sample movement (cantilever vibrations) producing rounded edges, the implementation of this process enables us to produce tips with sub 250 nm dimensions which retain their structural integrity.

**Figure S1:** Scanning electron micrographs demonstrating the procedure for plateau, chisel and double-tip fabrication. **a)** Micrograph captured while milling along the plane of the cantilever and while the tip is being detached from the probe. **b-c)** Completed plateau-tip in side view and orthographic view respectively. **d-e)** Fabricated chisel-tip and double-tip respectively.

**S2. Damage Evaluation**


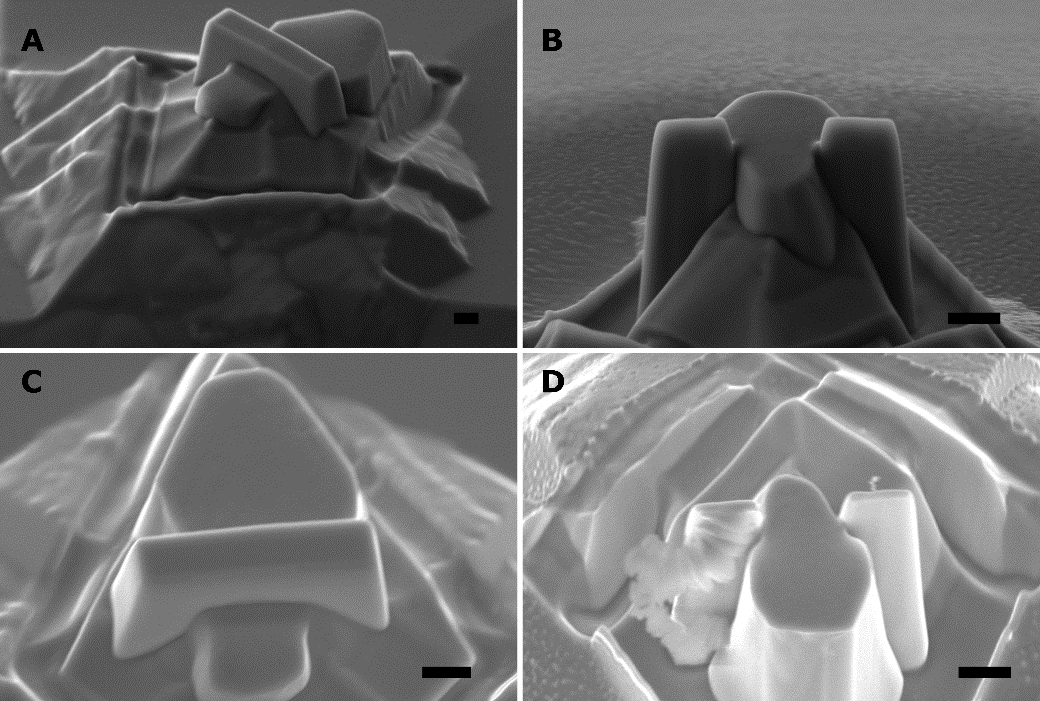
An important drawback of scanning probe technologies, whether for imaging or patterning lies in the relative short longevity of the tip. Commonly, the tip becomes either blunt or broken to some irregular shape after several scans or patterns depending on the application. This can be attributed to the extremely sharp apex which is typically between 10nm and 20nm and is therefore prone to damage due to shear and bending forces from the substrate. In using custom tip geometries with significantly larger cross-sections (l>250nm) we find that our probes can withstand considerably more patterning and imaging operations. Figures S2A and S2B show tips fabricated and imaged prior to use. The same tips are subsequently imaged using an SEM after patterning several mm^2^ of 250nm thick photoresist and can be seen in figures S2C and S2D. . While in some cases photoresist residue can be found on the tip, there is no observable structural deviation before and after use. We thereby conclude that the probes fabricated possess the added advantage of significantly increased longevity.

**Figure S2:** Low tip wear in nc-SPL using chisel-tips and double-tips. **a-b)** SEM micrographs captured before patterning **c-d)** SEM micrographs captured post patterning. It can be seen that while resist residue may be present on the probes, there is no perceivable damage on the probe.

**S3. Pattern Designer**

The following procedure is used in code in order to simulate the output of the lithography based on a given path followed by the cantilever. First the dimensions of the chisel or the double tip are defined and registered as a polygon as well as the separation between the two tips for the case of double tip lithography. Next, the simulation resolution is defined as the desired incremental distance along the path. By superimposing the locations of the chisel or double tips and joining them, the precise lithography output is predicted as shown in figure S3. Following this procedure and iterating over different directions, the dependence of the patterned linewidth on the patterning direction is determined.


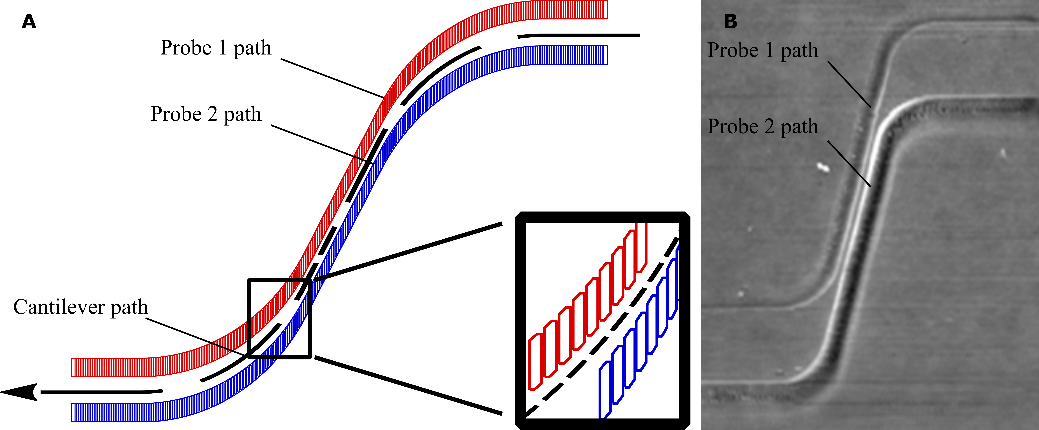


**Figure S3:** Pattern designer used to simulate the lithography output. **a)** Annotated simulation output **b)** AFM micrograph of resulting patterns.

**S4. Pattern Transfer**

Here we demonstrate the ability to transfer patterns from a resist layer to an SOI wafer. The SOI wafer was spun using a solution of Poly(methyl methacrylate) PMMA 495k 4% in anisole at 6000rpm and baked for 10 minutes at 180^o^C, producing a uniform resist layer of 250nm. Subsequently the wafers were patterned using a chisel tip with 1μΝ at a patterning speed of 10 μm/s producing the lithography output of figure S4 a). The patterns were then etched via RIE for 30 seconds using 100ccpm CHF_3_ at 200W. Finally, the remaining PMMA was removed by immersion in acetone at 60^o^C and sonication. Figure S4a demonstrates that post patterning the resist is located at either side of the track produced by the tip. After pattern transfer is completed and the resist has been removed from the surface, all the residual resist is dissolved and removed (figure S4b). The lithography result after pattern transfer can be seen in figure S4b.


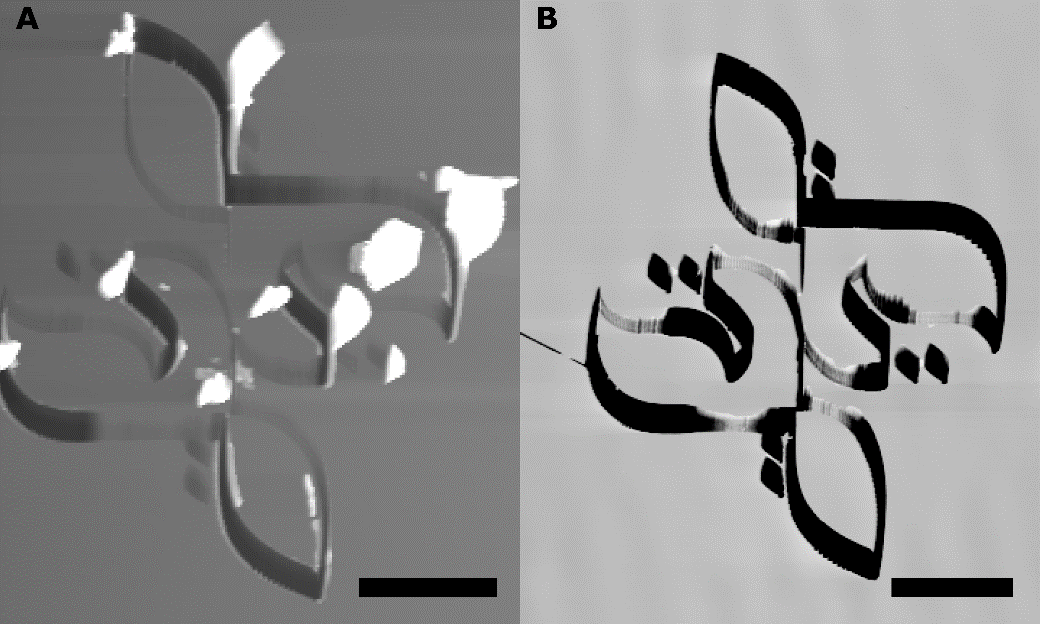


**Figure S4:** Pattern transfer to from PMMA resist layer to SOI wafer. **a)** AFM micrograph of patterns produced directly on PMMA **b)** AFM micrograph of patterns transferred to SOI wafer via RIE and subsequent stripping of PMMA. Scale bars are 5μm.

**S5. Effect of Probe Geometry to Linewidth**


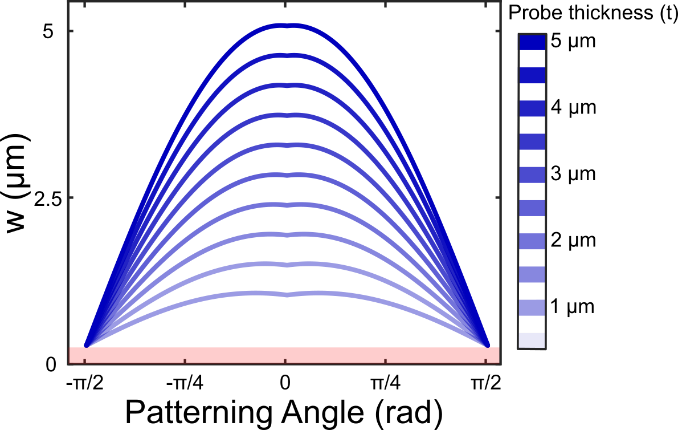
Here we simulate the dependence of the linewidth to the dimensions of the probe and the patterning angle. The minimum width of a patterned line is limited to the smallest dimension of the chisel tip which is normally the width. The length of the tip defines the largest linewidth which can be produced. In figure S5 the solid lines represent to a different chisel lengths between 0.5μm and 5μm scanned angles spanning -π/2 to π/2 radians with a fixed thickness of 0.25μm.

**Figure S5:** Simulated linewidth as a function of the width of the chisel-tip (w) and the patterning angle. Reducing the linewidth can be achieved by decreasing the width of the probe as in conventional SPL but also by controlling the patterning angle. The minimum width for chisel-tips is defined by the minimum dimension of the tip.

**S6. Section Height and Pattern Roughness**

Figure S6 shows a section of the patterns presented in figure 2E. We demonstrate that the etch depth during pattern transfer is 185nm. The roughness of the patterns is found to be 0.4nm and is evaluated over a 1μm^2^. This value compares well with the roughness of 0.13nm for areas which were not patterned. Here, the small increase in roughness is as a result of the pattern transfer in the RIE and not due to the patterning operation.


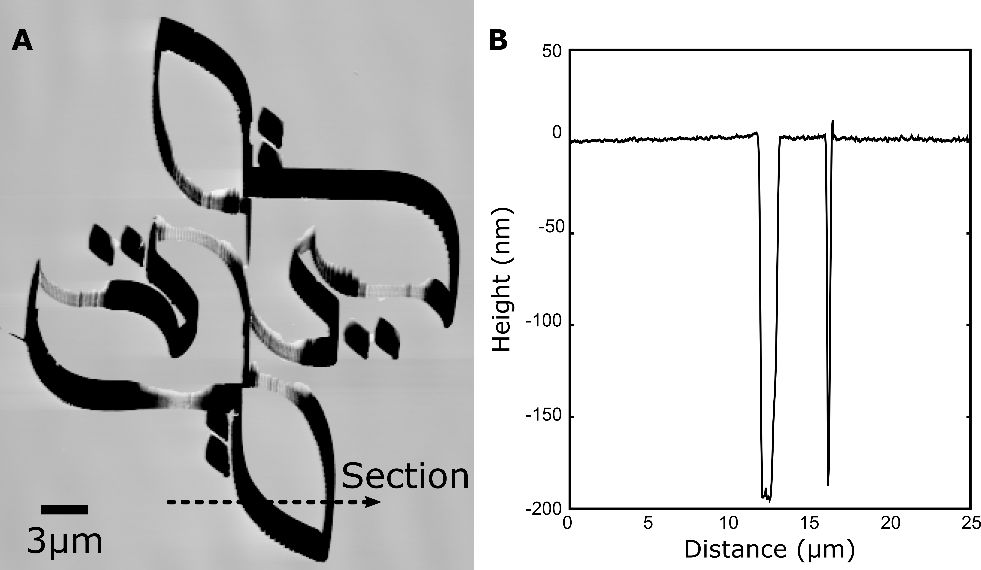
.

**Figure S6:** Pattern transfer from PMMA to silicon **a)** AFM micrograph of patterns etched in silicon **b)** Section of micrograph in a) showing the height distribution.
